# Implementing ctDNA Analysis in the Clinic: Challenges and Opportunities in Non-Small Cell Lung Cancer

**DOI:** 10.3390/cancers12113112

**Published:** 2020-10-24

**Authors:** Elisa Gobbini, Aurélie Swalduz, Matteo Giaj Levra, Sandra Ortiz-Cuaran, Anne-Claire Toffart, Maurice Pérol, Denis Moro-Sibilot, Pierre Saintigny

**Affiliations:** 1 Thoracic Oncology Unit, CHU Grenoble-Alpes, 38700 Grenoble, France or elisa.gobbini@hotmail.it (E.G.); mgiajlevra@chu-grenoble.fr (M.G.L.); atoffart@chu-grenoble.fr (A.-C.T.); dmoro-sibilot@chu-grenoble.fr (D.M.-S.); 2 Univ Lyon, Université Claude Bernard Lyon, INSERM 1052, CNRS 5286, Centre Léon Bérard, Centre de Recherche en Cancérologie de Lyon, 69373 Lyon, France; Sandra.ORTIZ-CUARAN@lyon.unicancer.fr (S.O.-C.); 3 Department of Medical Oncology, Centre Léon Bérard, 69373 Lyon, France; aurelie.swalduz@lyon.unicancer.fr (A.S.); maurice.perol@lyon.unicancer.fr (M.P.)

**Keywords:** liquid biopsy, circulating tumor DNA, next-generation sequencing, lung cancer, biomarker

## Abstract

**Simple Summary:**

The clinical relevance of tumor genotyping through analysis of ctDNA is now widely recognized at all steps of the clinical evaluation process in metastatic non-small cell lung cancer (NSCLC) patients. ctDNA analysis through liquid biopsy has recently gained increasing attention as well in the management of early and locally advanced, not oncogene-addicted, NSCLC. The aim of this review is to summarize the landscape of liquid biopsies in clinical practice and also to provide an overview of the potential perspectives of development focusing on early detection and screening, the assessment of minimal residual disease, and its potential role in predicting response to immunotherapy.

**Abstract:**

Tumor genomic profiling has a dramatic impact on the selection of targeted treatment and for the identification of resistance mechanisms at the time of progression. Solid tissue biopsies are sometimes challenging, and liquid biopsies are used as a non-invasive alternative when tissue is limiting. The clinical relevance of tumor genotyping through analysis of ctDNA is now widely recognized at all steps of the clinical evaluation process in metastatic non-small cell lung cancer (NSCLC) patients. ctDNA analysis through liquid biopsy has recently gained increasing attention as well in the management of early and locally advanced, not oncogene-addicted, NSCLC. Its potential applications in early disease detection and the response evaluation to radical treatments are promising. The aim of this review is to summarize the landscape of liquid biopsies in clinical practice and also to provide an overview of the potential perspectives of development focusing on early detection and screening, the assessment of minimal residual disease, and its potential role in predicting response to immunotherapy. In addition to available studies demonstrating the clinical relevance of liquid biopsies, there is a need for standardization and well-designed clinical trials to demonstrate its clinical utility.

## 1. Introduction

The utility of tumor genotyping is widely recognized at all stages of the clinical evaluation process in non-small-cell lung cancer (NSCLC) [1]. The deeper understanding of specific genomic alterations driving NSCLC development led to novel therapeutics with a dramatic impact on patients’ outcomes [2]. Recently approved drugs will certainly have a further impact on outcomes in the near future. Molecular profiling is equally essential at the time of acquired resistance to optimize subsequent treatments in the oncogenic-driven population [3,4]. 

While tissue-based molecular analysis is the gold standard for precision medicine, liquid biopsies have recently gained increasing attention due to the approval of EGFR tyrosine kinase inhibitors administration on ctDNA-based mutation detection. ctDNA analysis has also been shown to be feasible to evaluate the amount of molecular alterations characterizing the tumor by the so-called blood tumor mutational burden (bTMB). bTMB has recently been approved by the US Food and Drug Administration (FDA) as a first-line predictive biomarker of response to the combination of chemotherapy and immunotherapy. Despite its questionable positive predictive value, bTMB remains an important variable to consider in exploratory studies designed to identify integrated predictive biomarkers in the immuno-oncology field.

Other potential applications of ctDNA analysis are emerging in different settings of lung cancer management, such as lung cancer screening. Literature data are still scarce, but the use of ctDNA for screening is attractive to avoid the risk related to radiological assessments along with their limitations [5,6,7]. Likewise, monitoring of minimal residual disease (MRD) after a surgical treatment may help patient selection in the adjuvant setting [8,9]. In this context, liquid biopsy could address the risk of relapse, helping to tailor adjuvant strategies.

Finally, liquid biopsy could provide interesting insights into response to systemic therapies. Several studies have suggested a correlation between the dynamic modification of ctDNA over time and the objective response to targeted agents or immunotherapy [10,11,12,13,14,15,16,17]. The duration of immunotherapy in long responder patients after two years of treatment is debated. ctDNA could be useful in this context to differentiate patients whose disease was cleared or not by the treatment and offer them different treatment strategies [18].

**Figure 1:**
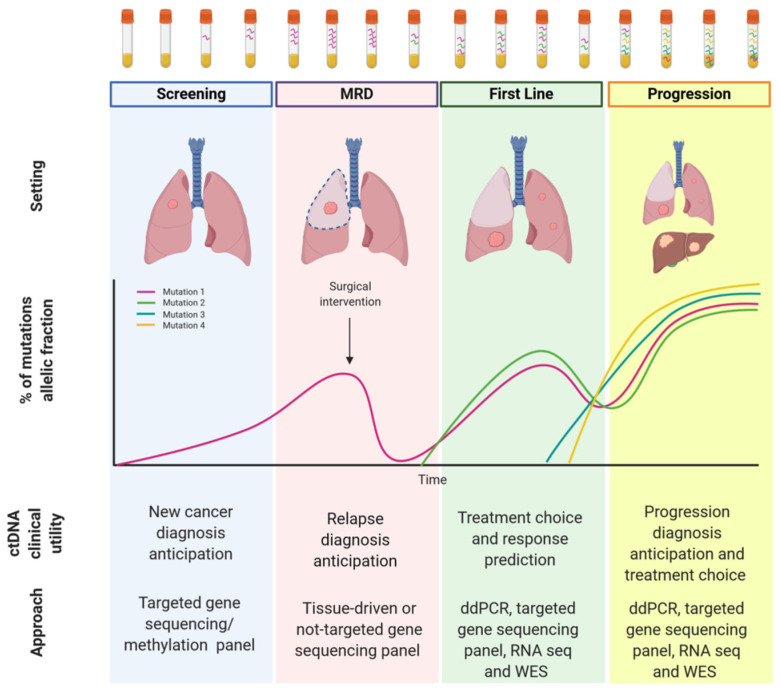
Circulating tumor DNA (ctDNA) clinical application in Non-Small-Cell Lung Cancer.

In this review, we will go through the landscape of liquid biopsy in NSCLC patients (Figure 1), describing technical challenges and clinical applications in routine practice. We will also provide an overview of the potential perspectives of liquid biopsies in the setting of screening, MRD assessment, and immunotherapy.

## 2. Technical Issues for Routine Practice

### 2.1. ctDNA in Liquid Biopsy

Liquid biopsy usually implies a simple peripheral blood draw, capturing a wide variety of soluble factors, including circulating DNA but also RNA, biomarker proteins, circulating tumor cells (CTCs), and exosomes. Cell-free DNA (cfDNA) includes a wide group of DNA coming from healthy tissue, inflammatory cells, and tumor cells following apoptosis, necrosis, or active release. It has a half-life of 16 minutes and is cleared through the liver and kidneys [19]. Levels of cfDNA are higher in cancer patients and include cfDNA shed by tumor cells, which is known as ctDNA [20,21]. Although cfDNA and ctDNA are both measurable in blood samples, detection of ctDNA is clinically more relevant than cfDNA, which is derived from tumors as well as non-tumor sources [22]. ctDNA represents <0.01% to 10% of cfDNA and its amount depends on several factors including the nature of the primary tumor, tumor grade and vascularity [21,23,24], physiological clearance and degradation [25,26,27], rate of release, cell status [24,27], time of blood draw, and therapy [21,28,29]. ctDNA can be released into the blood-stream from primary tumors, CTCs, and metastases, and it is usually highly fragmented (35 to > 10000 bp) [19,23,30,31]. Since ctDNA content can be influenced by several clinical factors, these are crucial to be considered when interpreting liquid biopsies results in clinical practice. Besides these clinical considerations, pre-analytical and analytical aspects are of amount importance and still need to be standardized.

### 2.2. Technical Considerations for the ctDNA Use

The main challenge using liquid biopsy is the detection of a minor allelic fraction of mutant DNA on fragmented cfDNA requiring optimization of pre-analytical, analytical, and post-analytical (patient- and tumor-related characteristics) aspects. These different steps have to be well controlled to ensure reliable results and to allow optimized clinical decisions. 

#### 2.2.1. Pre-Analytical Aspects

##### Source

According to the joint American Society of Clinical Oncology (ASCO) and College of American Pathologists (CAP) review, the optimal sample type for obtaining ctDNA is plasma rather than serum [32]. Indeed, leukocyte-derived normal DNA, which dilutes the ctDNA, is lower in plasma fraction. 

##### Tubes

The optimal types of tubes for the collection of are EDTA anticoagulant or cell (leukocyte)-stabilizing tubes (such as Roche, Strek, PAXgene, Arios) [33,34,35]. Importantly, samples must be processed within six hours when using EDTA tubes in order to avoid the risk of white blood cell lysis, which could dilute the ctDNA fraction. Conversely, the time for tube processing ranges from 48 h to 5 days at room temperature with cell-stabilizing tubes, allowing more flexibility for transportation. 

##### Plasma Extraction

Plasma is collected from whole-blood with a two-step centrifugation protocol in order to ensure cell-free plasma and limit genomic DNA contamination from whole blood cells. A first centrifugation at 1200 to 1600× *g* for 10 min is performed to remove cells. After supernatant harvesting, plasma is collected and centrifuged again at 3,000 to 16,000× *g* for 10 min (high-speed centrifugation) to remove all cellular-derived contaminants [36,37,38]. 

##### Storage and ctDNA Extraction

For future ctDNA extraction, processed plasma should be aliquoted into single-use fractions and stored at −20 to −80 °C for up to nine months or in liquid nitrogen to extend the storage life [39]. Repeated freeze/thaw should be avoided. The extraction process is a crucial step, and many different methods and protocols are reported, which may influence ctDNA yield and purity. Studies directly comparing different ctDNA isolation methods are lacking. However, a recent experience has found comparable ctDNA yields when using automated and manual ctDNA purification methods [40], suggesting that automated platforms may help in routine practice [32].

#### 2.2.2. Analytical Aspects and Methods of ctDNA Analysis

Ideally, ctDNA assay development should include controls for cfDNA purification efficiency, leucocyte DNA contamination, and cfDNA quantity [22]. There are various types of ctDNA assays, and all have different performances in terms of assay robustness and limits of detection for the various genomic alterations [32]. The genomic coverage (i.e., targeting specific genomic alterations versus broader panels of genes or a combination of these approaches) should also be considered when choosing a ctDNA assay [22]. ctDNA genotyping can be targeted to known mutations in predefined genes or non-targeted and aim at whole exome screening (discussed below) [41]. Targeted approaches are generally more sensitive than non-targeted assays; however, ultrasensitive technologies have been described [19]. 

##### PCR-Based

Current ctDNA profiling methods include PCR-based methods that allow the detection of hotspot mutations on selected genes (i.e., *EGFR T790M* point mutations in patients relapsing under first- or second-generation EGFR tyrosine kinase inhibitors (TKI) or *G1202R ALK* resistance mutation to better determine the sequence of TKI in patients with *ALK*-rearrangement). Among them, two main approaches are associated with high-sensitivity: droplet digital polymerase chain reaction (ddPCR) and BEAMing. ddPCR consists of the emulsification of extracted ctDNA, master mix, and Taqman reagents in droplets in order to isolate a single DNA molecule in each droplet. Two colors can be distinguished, the target mutant codon of interest and the conserved sequence, defining negative or positive droplets, respectively. One hotspot mutation is evaluated in each well, but multiplexing approaches are also developed that allow the combined detection of several mutations. This approach allows absolute quantification with high-sensitivity (0.01–0.001%). Beaming involves PCR-amplified DNA molecules binding to magnetic beads through an emulsion-PCR to detect specific mutations. Beaming does also allow absolute quantification. PCR-based methods are not recommended for the detection of gene-fusion [1]. Moreover, new generation-TKIs are associated with an increasing level of tumor heterogeneity and involve multiple mechanisms of resistance, and even if PCR-based methods can be useful for selective applications, they will probably gradually be replaced by next-generation sequencing (NGS) approaches, with broad coverage, simultaneously interrogating multiple genes and identifying a broad range of molecular alterations, including chromosomal rearrangements.

##### NGS-Targeted Approaches

Not all NGS assays are equivalent in terms of limits of detection, gene panels, and numbers of detectable variants; therefore, different assays should not be assumed as interchangeable. Basic NGS workflow is designed to detect mutations above a minimum of 0.1% to 1% mutant allele fraction [28]. Two targeted approaches can be distinguished: amplicon-based assay and hybrid-capture based assay. The amplicon-based assay consists of the amplification of short amplicons with specifically-designed primers followed by high-throughput PCR performed on a NGS platform. InVisionFirst™, the eTamSeq assay from Inivata, is a 36-genes panel related to lung cancer. It had been compared to ddPCR and had demonstrated high sensitivity for the detection of point mutations, indels, and high concordance with ddPCR with 95% positive predictive value and 100% of positive percentage agreement [42]. Hybrid-capture based assays are based on DNA hybridization to a DNA capture via molecular inversion probe. Different panels are available, including, for example, FoundationACT™, Guardant 360^®^, and Avenio^®^. The FoundationACT™ assay was compared to tissue using FoundationOne^®^ on patients with colorectal cancer and demonstrated high sensitivity and specificity [43]. Guardant 360^®^ was also used to compare results obtained on paired tissue and plasma samples of NSCLC patients with molecular alterations and showed 92 to 100% positive predictive value [4]. CAPP-Seq or cancer personalized profiling by deep sequencing is another targeted approach based on a patient tumor mutation analysis. It combines optimized library preparation methods for low DNA input masses with a multi-phase bioinformatics approach to design a “selector” consisting of biotinylated DNA oligonucleotides that target recurrently mutated regions in the cancer of interest. To monitor ctDNA, the selector is first applied to tumor DNA to identify cancer-specific genetic aberrations of a given patient that will then be used to circulating DNA to quantify them. It is theoretically associated with a detection limit of 0.00025% mutant alleles [44]. 

##### Whole-Exome Sequencing

While whole-exome sequencing (WES) on ctDNA has been shown to be feasible [45], allowing determination of the mutational tumor burden and the entire DNA coding sequence, it is associated with lower coverage as compared to limited gene panels and increased complexity of analysis and cost.

### 2.3. Liquid Versus Tissue Biopsies

**Figure 2:**
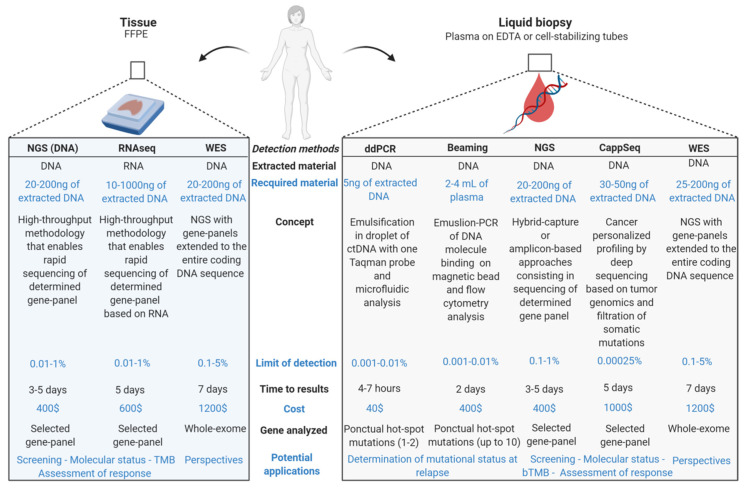
Tissue versus liquid biopsy: methods of molecular characterization. bTMB: blood tumor mutational burden, whole-exome sequencing (WES).

The most obvious benefits associated with the use of liquid biopsy are the limited invasiveness and reduced procedural risks for the patient compared with the collection of tumor specimens [46]. Whilst solid tumor biopsies provide a snapshot of the specific region of origin; a liquid biopsy may offer a more inclusive representation of the whole tumor burden, including intra-tumor subclones and metastases. On the other hand, the information provided by liquid biopsy cannot be directly correlated with the histological characteristics of the tumor [32]. An approach was proposed by Moss et al. to overcome this problem [47], using a method based on the deconvolution of cfDNA methylation using a reference methylation atlas of 25 human tissues and cell types. The authors were able to identify the tissue of origin of cfDNA derived from healthy individuals, transplant recipients, patients with sepsis, and patients with metastatic cancer or cancers of unknown primary [47]. As already highlighted earlier, concordance between tumor tissue- and blood-based assays is influenced by several factors. Tumor type and metastatic sites, stage of disease, tumor heterogeneity, time of sampling, and clonal versus subclonal variants can influence assay detection capacities independently of analytical factors [22,32]. Notably, ctDNA yields are lower at the early stages of cancer and non-detectable for certain types of cancers like neoplasms of the central nervous system, which can limit the utility of ctDNA in these cases [23,48]. For patients with advanced disease, ctDNA test results appear more reliable when performed at disease progression rather than during response to therapy [32]. This implies that confirmation of ctDNA negative tests is still recommended using tumor biopsy [1,41,49]. At the other end, false-positive findings can be introduced due to the presence of cfDNA from multiple sources, including age-related mutations from hematopoietic cells that and ctDNA mutations that are shared between cancer and benign conditions [41]. In conclusion of this section, tissue and liquid biopsies may be considered as two complementary methods, and clinical aspects are crucial to take into account when interpreting ctDNA results [50,51,52,53,54] (Figure 2).

## 3. Evidence for the Clinical Relevance of Liquid Biopsy in Routine Practice

The ASCO and CAP [32] highlighted the lack of well-conducted studies validating the clinical utility of liquid biopsy. However, compiled data obtained in the field of oncogenic addiction in NSCLC tend to suggest that liquid biopsies are clinically relevant and an interesting tool for treatment guidance. 

The implementation of molecular testing at the time of diagnosis in patients with advanced stage and the administration of molecular targeted agents nearly doubles the median of overall survival (OS) of patients with oncogenic-driven NSCLC. Moreover, it prevents the use of ineffective immunotherapies in this subgroup of patients [55]. However, an exhaustive molecular portrait by using a gene sequencing panel or by searching only specific molecular alterations of interest for the first-line prescription is quite tissue-consuming. Large studies revealed that 25% to 30% of NSCLC patients are treated without knowing the *EGFR*- and *ALK*-status and even more with no information about other molecular alterations potentially treatable with a targeted agent [56]. Moreover, a tissue biopsy is an invasive approach, with a 19% adverse event rate reported [57] and a mortality rate of 1% [58]. Finally, a re-biopsy is frequently unfeasible or too demanding for the patient. Liquid biopsies have the potential to overcome these issues in the routine care of patients. 

Two aspects seem to be relevant when regarding the use of liquid biopsy in treatment decision: the performance associated with the assay (robustness, reproducibility, sensitivity, and specificity) and the correlation with clinical outcomes. 

In the context of NSCLC, to be performant, an assay must be able to assess point mutations (*EGFR-, BRAF* V600E, *ERBB2,* and *KRAS* G12C mutations), exon skipping mutations (*MET* exon 14), and fusions or fusion transcripts (*ALK*-, *ROS1*-, *RET*-, *NTRK*-, and *NRG1-*rearrangements). Using NGS targeted gene panels, tissue- and plasma-analysis has demonstrated high concordance rates for each molecular alteration considered [4]. Moreover, some studies demonstrated that adding ctDNA analysis at the time of diagnosis improved molecular alterations detection in a significant proportion of patients [59,60]. In the NILE trial, the addition of ctDNA to tumor tissue characterization improved the number of patients who benefited from targeted therapies by 48% together with a shorter median turnaround time (9 days for ctDNA analysis versus 15 days with tissue-based analysis) [61]. 

More importantly, several studies also suggested that the majority of patients who received targeted therapies based on blood-based tests achieved objective complete or partial responses. Remon et al. performed the Inivata InVision^TM^ assay in 81 advanced NSCLC patients. All classes of molecular alterations detected were able to predict response to targeted therapies with a disease-control rate and median progression-free survival (PFS) comparable to those expected with a tissue-based test [62]. The BENEFIT trial was focused on 183 EGFR positive Asian NSCLC patients receiving gefitinib as a first-line treatment [63]. The study explored the predictive value of blood-based EGFR mutation detection by a 168-genes NGS panel reporting an overall response rate of 72% and a PFS of 9.5 months. In another study, Remon et al. focused on 48 *EGFR*-mutated patients receiving osimertinib as a second-line treatment after a 1^st^ generation EGFR tyrosine kinase inhibitor. The authors evaluated the correlation between outcomes and T790M status determined by using the blood-based Inivata InVision^TM^. ctDNA-based T790M mutation was detected in 50% of the study population with a partial response rate of 62.5% and a disease control rate of 90%. Six- and 12-months PFS were 66.7% and 52%, respectively. [64]. These results were comparable to those obtained by a tissue-based test showing that ctDNA-based EGFR mutation detection can be used to guide EGFR tyrosine kinase inhibitor prescription. 

Moreover, while patients with oncogenic-driven NSCLC usually present a dramatic objective response to targeted therapies, they eventually all develop acquired resistance. Several acquired resistance mechanisms have been observed: (i) secondary mutations on the oncogenic driver overcoming the tyrosine kinase inhibitor activity (*EGFR* T790M or C797S, and *ALK* G1202R mutations); (ii) tyrosine kinase inhibition through by-pass mechanisms such as point mutations (*KRAS*-, and *BRAF*-mutations), amplifications (*MET*-amplification is the most frequent mechanism of resistance for *EGFR*-mutated patients with progressive disease on osimertinib as first-line therapy) and fusions (*RET*- and *BRAF*-fusions); and (iii) histological transformation. Because the mechanism of resistance drives the second-line strategy, ctDNA appears as a clinically relevant tool in patients with progressive disease, even if tissue remains the better way to evaluate histological transformation. However, it is important to note that, compared to diagnosis, ctDNA analysis at the time of disease progression may be challenging. Indeed, ctDNA quantity in plasma is lower, resulting in decreased sensitivity [65]. For example, brain metastases release ctDNA into the surroundings and cerebrospinal fluid, but the blood-brain barrier limits ctDNA to enter the peripheral blood-stream. In those cases, the analysis of cerebrospinal liquid represents an alternative liquid biopsy [66,67]. 

Despite these limitations, the feasibility and value of molecular profiling on ctDNA at the time of progression is established. AURA3 study compared osimertinib to chemotherapy in T790M-positive patients progressing after first- or second-generation EGFR tyrosine kinase inhibitors. Ancillary studies demonstrated similar objective response rates and PFS when the T790M mutation was detected on plasma and on tissue [65]. Moreover, patients who are plasma-positive but tissue-negative for T790M were shown to benefit from osimertinib [68]. In the FLAURA study, exploring the efficacy of osimertinib as an upfront treatment in EGFR-positive patients compared to a first-generation tyrosine kinase inhibitor, ctDNA analysis with a NGS panel allowed the identification of a large panel of resistance mechanisms including point mutations, fusions, and amplifications, some of which were potentially targetable [49]. 

Likewise, other oncogene-addicted NSCLC patients progressing under ALK or ROS1 tyrosine kinase inhibitors were shown to benefit from the optimization of subsequent treatment strategies based on ctDNA characterization [42,65,69]. 

## 4. Lung Cancer Screening 

Despite recent advances in the treatment of advanced stage, lung cancer remains the leading cause of cancer-related death worldwide [70]. Indeed, lung cancer is more often diagnosed at an advanced stage, not allowing treatment with curative intent [71]. Radiological screening programs with low-dose computed tomography (LDCT) are currently recommended in a high-risk population of patients defined by their smoking history. Large randomized studies have shown a considerable reduction in lung cancer-related mortality with the LDCT-based screening [58,72]. However, the risk of over-diagnosis and the lack of consensus in the target population have slowed down the widespread application of this strategy. Moreover, no early detection approaches have been proposed for non-smokers and, given the increase in non-smoking related NSCLC [71], this represents a substantial unmet need. 

Liquid biopsy could be interesting in this setting as it would avoid the risk of second radiation-related cancers and allow longitudinal surveillance by using a non-invasive method. However, the costs and the low sensitivity of the technologies currently available for the identification of early disease (47–50% sensitivity for I stage cancers) [23,44] limit its development in this setting. Increasing the number of target mutations contained in the gene-sequencing panel may increase the sensitivity of the test, however, increasing the cost-effectiveness issues. 

Another strategy could be to look at aberrant methylation of certain regions of ctDNA as a biomarker for early diagnosis. Indeed, methylation of genes such as *MGMT, RAR-b, RASSF1A, p16INK4a,* and *DAPK* have been reported in the serum DNA of lung cancer patients and can be considered as a tumor marker [73,74,75,76,77]. Unfortunately, the frequency of these methylations at a single locus is too low to be sufficient for diagnostic purposes. Some studies have, therefore, considered the possibility of combining the detection of multiple methylation markers in order to increase sensitivity [75,76]. For instance, Hsu et al. used a panel of six methylation markers (*BLU*, *CDH13*, *FHIT*, *CDKN2A*, *RARβ*, and *RASSF1A* genes) on plasma samples from 63 lung cancer patients compared and 36 cancer-free individuals [76]. The individual relative association for lung cancer ranged from 1.4 to 10.2 for a given methylated gene in patients compared with controls. Moreover, diagnostic value increased when more than two markers were hypermethylated, with 73% and 82% sensitivity and specificity, respectively. However, this study included both patients with early and advanced-stage disease that may result in an overestimation of the assay sensitivity. 

Modern screening tests combine multiple blood markers to increase sensitivity. CancerSEEK is a blood test combining cell-free DNA (cfDNA)-based tumor-associated mutation and eight circulating proteins for early disease detection of several cancers [5]. The study included 104 stage I to III lung cancer patients in which the test had a 59% sensitivity. Similar results were provided by a cfDNA-based blood test merging information from targeted sequencing, whole-genome sequencing, and methylation analysis of serum samples from 73 lung cancers [7].

Considering these results and the low ctDNA concentration before clinical evidence of disease, it is at this time challenging to validate a ctDNA-based screening test, and in the future, more studies are needed.

## 5. Minimal Residual Disease Detection

It is well known that the benefit of adjuvant chemotherapy for patients with early-stage NSCLC translates into an absolute 5-year survival advantage of 4% to 5.4% suggesting this strategy to be of interest in a small proportion of patients [78]. To date, there are no biomarkers that can identify high-risk patients, and clinicians rely solely on the TNM classification and comorbidities. The possibility of having one or more biomarkers able to identify the patients most at risk of relapse, but especially those who do not need complementary treatments, is, therefore, particularly important. 

ctDNA may serve these purposes by identifying MRD still present after initial treatment and potentially responsible for disease relapse or recurrence. Furthermore, the detection of this biomarker could provide useful information to tailor therapy. In fact, the treatment could be adapted to the characteristics of the residual tumor cells after the initial treatment for a personalized strategy. Finally, sampling patients longitudinally would allow the early detection of a change in the amount of ctDNA and could translate into therapeutic anticipation compared to traditional imaging follow-up. 

Two different approaches have been proposed for MRD detection according to tumor tissue availability. In the first one, resection specimens or tumor biopsies are analyzed to identify a limited panel of mutations present in the primary tumor that will be used for ctDNA detection. This method is more sensitive than the non-targeted sequencing (<0.01%) but requires detailed information for each patient [79]. Also, this approach may miss additional genetic alterations acquired by cancer cells constitutive of MRD. Such information is not required in the non-targeted approach that makes use of a large panel of mutations known to be associated with lung cancer. This latter approach is associated with a lower sensitivity (above 1–5%), limiting its utility in patients without metastatic disease at baseline. 

Several studies have suggested a role of ctDNA-based MRD detection in lung cancer by documenting marked differences in presurgical and postsurgical levels of ctDNA [80,81,82]. Subsequently, two studies have simultaneously investigated the role of ctDNA for the early detection of NSCLC relapse [8,9]. In the TRACERx trial, primary tumors were analyzed by whole-exome sequencing, and a bespoke multiplex-PCR assay panel of single nucleotide variants for each patient was defined [9]. ctDNA monitoring was then performed longitudinally after the surgical intervention, defining a positive result as the detection of at least two predefined variants. The study showed that the ctDNA postsurgical detection correlated with disease recurrence. Indeed, 13 of 14 patients who experienced disease relapse, and only 1 of 10 patients who did not, had measurable ctDNA before or at the time of clinically evident recurrence. Overall, the ctDNA detection anticipated the radiological diagnosis by a median interval of 70 days.

Likewise, Chaudhuri et al. demonstrated that postsurgical ctDNA detection correlated with relapse in a cohort of 37 stage I to III NSCLC [8]. In this study, authors used a CAPP-seq panel of 128 genes recurrently mutated in lung cancer. ctDNA was detectable in 20 of 37 patients in at least one time-point after the surgical intervention, and all of them eventually relapsed. The ctDNA becomes measurable before the radiological diagnosis of progression in 72% of patients, with a median interval of 5.2 months. Patients with detectable ctDNA on a sample collected less than four months after surgery had a significantly lower relapse-free survival and OS than those for whom ctDNA was undetectable. 

However, several limitations have to be taken into account. Further technical improvement is needed considering the low rate of ctDNA available in early-stage lung cancer and the cost-effectiveness of these technologies. Moreover, a consensus on the number of mutations to be necessarily identified to avoid false-negative results has not yet been reached. Clonal mutations present in the primary disease may not be represented in metastatic seeding, and it is not known if the ctDNA assay can capture all the ctDNAs present in the blood or only a specific subset. On the other hand, false-positives may be due to aging or benign diseases [83,84] and have to be considered. Finally, while clinically relevant, these studies do not demonstrate the clinical utility of detecting MRD and treating recurrent disease earlier. This will require well-designed studies to demonstrate improved OS over the absence of MRD detection.

## 6. Evaluation of Response to Targeted Therapies 

Targeted therapies typically result in a high response rate and durable responses; however, some patients experience disease progression earlier than expected, probably due to primary or acquired resistance mechanisms. ctDNA dynamics has been evaluated as a predictive biomarker to identify these patients. 

Buttitta et al. evaluated ctDNA dynamics in plasma of EGFR-mutated patients harboring a T790M mutation and treated with osimertinib as second-line therapy [10]. Sixty-four patients were strictly monitored during the first month of therapy, and plasma was analyzed by using the EGFR Cobas test, showing a substantial decrease in sensitizing EGFR mutant allele down to not detectable value in 89% of cases. The remaining 11% of patients were defined as plasmatic poor responders as the EGFR mutation was still detectable at the end of the first month of treatment. Plasmatic poor responders achieved disease progression in five (71%) cases, stable disease in two (29%) cases as best response, and experienced a significantly shorter median PFS (4.3 months) as compared with plasmatic good responders (13.3 months). Likewise, ctDNA level modification over time in *EGFR*-mutated patients was evaluated in the AURA3 study [11]. Early clearance of *EGFR* mutations, assessed by the Guardant360 test or a ddPCR, after three weeks of treatment with osimertinib was associated with a longer PFS (10.9 vs. 5.7 mo) and a higher ORR (81% vs. 50%). 

Similarly, Kwon et al. evaluated the ctDNA dynamics in ALK-rearranged NSCLC patients receiving crizotinib and alectinib by using the Guardant360 panel [85]. Two months after treatment initiation, 55% of the study population had a complete blood clearance of ALK-fusion and/or relevant associated mutations present at baseline. Moreover, complete clearance was significantly correlated with improved outcomes in terms of PFS and OS.

In summary, plasma monitoring may be useful for early identification of early-progressive patients in the oncogenic-driven NSCLC setting. Indeed, emerging strategies associating chemotherapy and tyrosine kinase inhibitors could reduce early progressing patients but certainly add chemotherapy-related toxicity. ctDNA could be an interesting tool to evaluate which patients can better benefit from this combined strategy versus targeted monotherapies. 

## 7. ctDNA to Guide Immune Checkpoint Inhibitors Treatment Planning

Immune checkpoint inhibitors have completely changed the therapeutic algorithm for metastatic or locally advanced NSCLC [86,87,88,89,90,91,92,93]. However, it is clear that only a portion of patients achieve a long-term benefit from this strategy, and no sufficiently reliable biomarker for patient selection currently exists. Although high PD-L1 (≥50%) tissue expression allows the population to be enriched with patients who are more likely to respond, non-negligible clinical benefits can be found in patients with low or even negative PD-L1 [86,87,89,90]. In view of the cost of immunotherapy drugs and the long-term side effects they may cause, the identification of more reliable biomarkers is crucial to optimize the cost-effectiveness. 

In addition, with the advent of immunotherapy drugs, concepts such as pseudoprogression or hyper-progression have been introduced, accounting for 5–10% and 13–37% of patients treated with immune checkpoints, respectively, according to the case series and the definition used [94,95,96,97,98]. Biological markers to predict the long-term clinical benefit of these patients are urgently required to drive the treatment strategy. 

Below, we will describe the potential relevance of ctDNA-based assays for patients undergoing therapy with immune checkpoint inhibitors.

### 7.1. Baseline Identification of Responder Patients

TMB has recently received FDA approval for the prescription of immunotherapy in lung cancer patients.

High TMB, defined by various cut-offs of mutations per Mb, suggests the presence of more neoantigens released from tumor cells, increasing the chances of immune response activation and tumor-specific cytotoxic T lymphocytes triggering. TMB status can be assessed on tissue using whole-exome sequencing (WES) or targeted NGS of a more focused panel of genes [99,100]. Despite inconsistencies in the cut-offs definitions, high TMB appeared to be associated with greater clinical benefit (particularly ORR and PFS) among patients receiving immunotherapy for lung cancer [101]. However, the implementation of TMB assay into the clinic is hampered by several issues, including the tissue availability, which is limited to 34–59% of patients according to clinical trials [102,103,104]. Blood TMB (bTMB) can overcome this limitation, and several studies explored its predictive value. 

Inconsistent results can be found in the literature about the concordance between tissue and blood evaluation of TMB [105,106]. Indeed, the allele frequency of certain tumor-associated mutations can be low, and the sequencing depth is therefore crucial for the determination of bTMB. However, bTMB has been validated as a predictive biomarker of immune checkpoint inhibitor efficacy in NSCLC. 

Some studies retrospectively assessed the bTMB predictive value in advanced NSCLC patients receiving an immune checkpoint inhibitor as first or later lines of treatment [107,108,109]. They all used targeted genes panels (FoundationOne, Guardant OMNI, and NCC-GP15), adopting different cut-offs ranging from 6 to 20 mutations per Mb. bTMB was evaluable in about 70% of the study population, and it was defined as “high” in 26 to 56% of cases. Despite the heterogeneity in terms of panel used and cut-off definition, all these studies agreed about the bTMB positive predictive value. High bTMB patients achieved a longer progression-free survival (PFS) and a better overall response rate (ORR) once receiving immunotherapy compared to chemotherapy. Conversely, no difference or a better benefit from chemotherapy was observed in the low bTMB group. Of note, only one study reported an OS benefit according to the bTMB status [108]. In the MYSTIC trial (Guardant OMNI, a cut-off of 20 mutation per Mb), high bTMB patients in the durvalumab-tremelimumab subgroup showed a better OS compared to chemotherapy (21.9 versus 10 months). 

The first study prospectively validating the predictive role of bTMB was the B-FIRST trial enrolling advanced NSCLC patients to receive atezolizumab as an upfront regimen not-being selected for PD-L1 expression [110]. Seventy-eight percent of the study population was evaluable for bTMB FoundationOne test, and 23% of them were considered to have a high bTMB using a 16 mutation per Mb cut-off. High bTMB patients had a higher ORR (28.6% versus 4.4%), whereas no statistically significant differences in PFS and OS were detected. 

In view of these results, which are not completely satisfactory, as well as of the cost and the average results turnaround, bTMB has not yet entered into clinical practice. A significant amount of work is also required in terms of standardization. 

However, it is interesting to note that high bTMB was not associated with PD-L1 expression, suggesting that the two biomarkers may complement each other to select higher responder patients (109), but further prospective data are needed. 

### 7.2. Response Evaluation to Immunotherapy

The immune response triggered by immunotherapy induces an inflammation around the tumor, which can make the interpretation of radiological response challenging. Confirmation of a response or a progression requires a period of observation during which the patient still receives a treatment that most often does not work anymore. A biomarker able to discriminate between pseudoprogression and true progression would therefore be very useful. ctDNA-based assays have been investigated in this context in several small cohorts of cancer patients (less than 40) using different strategies [12,13,14,15,16]. In these studies, ctDNA levels were assessed by using various approaches, such as ddPCR (for patients with known mutations) or multigene NGS assay and TEC-seq, at baseline and during the immune checkpoint inhibitor treatment at different time points. Only in one study, paired blood and tissue samples were analyzed to select patients having mutations that were measurable in both the tumor tissue and plasma for the longitudinal evaluation [14]. They all concluded that patients in whom ctDNA levels decreased or became undetectable under treatment had favorable outcomes. Interestingly, in some cases, the ctDNA drop has even been associated with a better OS [13,15,16]. 

On the other hand, the increase in ctDNA levels may indicate a resistance to immune checkpoint inhibitors. Anagnostou et al. used a TEC-seq approach in 24 advanced NSCLC patients receiving an immune checkpoint inhibitor and 14 patients receiving nivolumab in the neoadjuvant setting [17]. Only genetic alterations identified in paired tissue and plasma samples were considered for the ctDNA analysis. Patients with clinical response had a complete clearance in ctDNA levels after initiation of therapy, whereas non-responders had no significant changes or an increase in ctDNA levels. Patients with initial response followed by acquired resistance had an initial drop followed by recrudescence in ctDNA levels. Patients without a molecular response had shorter PFS and OS compared to molecular responders. The molecular response was detected, on average, 8.7 weeks earlier than radiological imaging. Interestingly, authors were able to corroborate these results by the pathological evaluation of postsurgical specimens of the resectable cohort. 

According to these data, ctDNA-based response evaluation seems to be an interesting tool to associate with radiological assessment, but further data and prospective trials are needed. 

### 7.3. Identification of Completely Eradicated Disease in Long Responders 

Immunotherapy has led to the appearance of long responders in patients with metastatic disease. To date, no clear guidelines are available for these cases, and the treatment strategy is decided on a case-by-case basis. Having an idea of the amount of residual disease at this time point could be clinically useful in deciding to continue or stop immunotherapy. 

Recently, Hellman et al. hypothesized that ctDNA analysis of responders to immune checkpoint inhibitors may predict the long-term clinical benefit of this strategy [18]. This study included 31 advanced NSCLC patients still responding after at least 12 months of an immune checkpoint inhibitor representing 20% of the entire population. The authors used a CAPP-seq ctDNA profiling assay, either tumor-informed or not, according to the baseline tumor tissue availability. In patients with non-available tissue, plasma samples were considered positive for ctDNA if any mutation was detected. Plasma samples were collected longitudinally from six months after initiating treatment. ctDNA was not detected in 27 patients, 25 of whom did not progress. Conversely, all four patients who had measurable ctDNA at the surveillance time point eventually progressed after a median interval period of 4.4 months. Consequently, patients with undetectable ctDNA had significantly longer disease-free survival compared to those with measurable ctDNA. According to this study, ctDNA-based residual disease evaluation may be useful to distinguish between long-responder patients in those residual lesions contains viable cells versus those whose disease is completely eradicated. Whether treatment continuation in patients with detectable residual disease or treatment withdrawal in eradicated patients does alter their prognosis is unknown. 

## 8. Conclusions 

From this review, it is clear that liquid biopsy through the analysis of ctDNA will progressively be routinely used in the care of patients with NSCLC. This is already the case in patients with advanced disease when tissue available is limited and genomic analysis not possible. In many other situations, including response monitoring of patients with advanced disease treated with TKIs or immunotherapy and monitoring of MRD in patients with early stage of the disease, the use of liquid biopsies and ctDNA analysis is still not approved, but studies presented here demonstrated their clinical relevance. The two main challenges in the near future will be to first work on standardized assays and second to translate those interesting observations into clinically useful tests by performing well-designed clinical trials in different settings.
